# European General Practice Research Network (EGPRN): *Abstracts from the EGPRN meeting in Leipzig, Germany, 12–16 October 2016. Theme: ‘General practice/family medicine in a changing world’*

**DOI:** 10.1080/13814788.2017.1300653

**Published:** 2017-05-12

## Introduction to the theme ‘General practice/family medicine in a changing world’

Societies and healthcare systems in Europe are facing many changes: ageing, migration, increasing morbidity, shortage of financial resources. All these changes will influence the future care and research in general practice.

The ‘theme’ contributions are related with the following subtopics:

- General practice in a changing world (e.g. shortage of GPs, changing practice management)
- Innovations for future care and research (e.g. ambient assisted living, European networking)
- Primary care for patients suffering from chronic diseases
- The international development of professional and academic general practice
- The education and support of future general practitioners by innovative solutions

## References

[1] Koch K, Gehrmann U, Sawicki PT. Primärärztliche Versorgung in Deutschland im internationalen Vergleich. Ergebnisse einer strukturvalidierten Ärztebefragung [German Primary Care in International Comparison: Results of a Survey of Doctors]. Dtsch Arztebl. 2007;104:A2584–A2591. German.

[2] Kopetsch T. Dem deutschen Gesundheitswesen gehen die Ärzte aus! Studie zur Altersstruktur- und Arztzahlentwicklung [The German health care system is running out of physicians! Study on the development of age structure and number of physicians]. 5th ed Berlin: Kassenärztliche Bundesvereinigung; 2010. German.

[3] Nowossadeck E. Demografische Alterung und Folgen für das Gesundheitswesen [Demographic ageing and consequences fort he health care system]. Robert Koch-Institut Berlin; 2012 GBE kompakt, 3(2). Available from: http://www.rki.de/DE/Content/Gesundheitsmonitoring/Gesundheitsberichterstattung/GBEDownloadsK/2012_2_Demografischer_Wandel_Alterung.pdf?__blob=publicationFile

[4] Sachverständigenrat zur Begutachtung der Entwicklung im Gesundheitswesen. Bedarfsgerechte Versorgung – Perspektiven für ländliche Regionen und ausgewählte Leistungsbereiche (Gutachten 2014). Berlin; 2014 Available from: http://www.svr-gesundheit.de/fileadmin/user_upload/Gutachten/2014/SVR-Gutachten_2014_Langfassung.pdf

[5] Schneider A, Großmann N, Linde K; DFG Network Clincial Trials in General Practice The development of general practice as an academic discipline in Germany – an analysis of research output between 2000 and 2010. BMC Fam Pract. 2012;13:58. 22702476 10.1186/1471-2296-13-58 PMC3489844

[6] Steinhäuser J, Roos M, Haberer K, et al Bericht aus der Praxis: Das Programm Verbundweiterbildung plus des Kompetenzzentrums Allgemeinmedizin Baden-Württemberg-Entwicklung, Umsetzung und Perspektiven. Zeitschrift für Evidenz, Fortbildung und Qualität im Gesundheitswesen. 2011;105:105–109. German. 10.1016/j.zefq.2011.02.002 21496778

