# Supplementary material for: European General Practice Research Network (EGPRN): Abstracts from the EGPRN meeting in Leipzig, Germany, 12–16 October 2016. Theme: ‘General practice/family medicine in a changing world’
Source: Eur J Gen Pract. 2017 May 12;23(1):143–54. doi: 10.1080/13814788.2017.1300653 (PMC5774276; doi:10.1080/13814788.2017.1300653)
Supplement: Related texts

# Abstract: Challenges for general practice/primary care research in a changing world

**DOI:** 10.1080/13814788.2017.1300653

**Published:** 2017-05-11

**Authors:** Jan De Maeseneer

**Affiliations:** ^a^ Department of Family Medicine and Primary Health Care, Ghent University Belgium

Nowadays health systems are confronted with important challenges: there is the demographical and epidemiological transition: with an important increase of multimorbidity. There are the scientific and technological developments and especially the increasing impact of ICT in care. There are important cultural developments, presenting a new type of ‘patient’ (active, informed and critical). There is an increasing social health gap, caused by social determinants of health. All these global problems are present in the waiting room of the provider at the primary care level. These challenges require new types of questions. The components of primary care can be organized around the following clusters: proactive or pre-care; reactive care; chronic care; community/population-oriented care and health system components of primary care. For each of these clusters, we can formulate relevant research questions at the nano-, micro-, meso-, and macro-level.

Four dimensions may guide the conceptual basis of our research:

- **Equity**: e.g. research on access to care, including access to innovative care; financial accessibility; cultural accessibility;
- **Quality**: looking at structure-process and outcome, and evaluating the ‘medical/technical evidence’, the ‘contextual evidence’ and the ‘policy evidence’. Importantly, especially in the context of multimorbidity, our research requires a paradigm-shift from disease-oriented care, towards goal-oriented care to assess to what extent our interventions contribute to the achievement of the individual goals of the patient regarding quantity and quality of life;
- **Cost-effectiveness**: with a focus not only on ‘cost-reduction’ but on the creation of ‘added value’;
- **Sustainability**: the ‘sustainable development goals’ invite us to take an intersectoral look at health, involving welfare, work and education. The study of ‘community oriented primary care’ (COPC) and its contribution to ‘social cohesion’ is one of the strategies to look at ‘sustainability’.

From these research dimensions, a magnitude of research designs can be developed. Nowadays, we need to look at key concepts that contribute to innovative care: interprofessional cooperation, the concept of ‘social accountability’, the relevance of the care (‘does care matter for the patient/population?’), the contribution of care to social justice.

That kind of research projects, continuously developed in interaction with the populations we serve, will help general practice/primary care to contribute to health systems based on relevance, equity, quality, person- and people-centeredness, sustainability and innovation.

# Abstract: State of the art and development of family medicine/primary care in Germany. Report and comment on an EGPRN keynote

**DOI:** 10.1080/13814788.2017.1300653

**Published:** 2017-05-11

**Authors:** Tobias Deutsch, Thomas Frese

**Affiliations:** ^a^ Department of General Practice, Medical Faculty, University of Leipzig Leipzig Germany; ^b^ Institute of General Practice, University Halle-Wittenberg Halle/Saale Germany

On Saturday 15 October 2016, Prof Joachim Szecsenyi was the national keynote speaker at the 83th meeting of the EGPRN in Leipzig, Germany. He introduced the audience into the German healthcare system and current developments and challenges of the academic general practice in Germany. With this short paper, we want to report and comment this keynote session.

After a brief introduction into the German healthcare system (mandatory insurance, direct access to GPs and specialists, payment fee for service), Szecsenyi underlined the high workload of German GPs compared to other countries. On average, Koch et al., found a weekly work time of 51 hours and 243 patient contacts per week [1].

As a challenge of particular importance, he described current problems to maintain a sufficient GP workforce against the background of significant recruitment problems in general practice and an ageing population with increased demands on primary care supply [2,3]. There has been a dramatic shift in the GP–specialist ratio in the German ambulatory care in the last two decades. While the number of specialists increased by 57% between 1993 and 2012, the number of GPs decreased by 10% [4].

Subsequently, the role of the academic departments in strengthening general practice was emphasized. As a positive achievement of the last years it can be stated that, although funding is still insufficient in many cases, nearly every German medical faculty has a department or institute for general practice. The research output increased dramatically from 2000 to 2010 [5]. Furthermore, the GP associations are growing (e.g. German College of GPs (DEGAM)) and there is an increasing number of community-based GPs involved in innovative teaching and research. According to Szecsenyi, the current key tasks of the academic departments are attracting young physicians to the specialty, doing research on the core problems of the profession (e.g. reasons for encounter, prevention, chronic and end of life care, multimorbidity, prescribing, polypharmacy, patient safety, quality of care, shared decision making, risk communication, etc.); developing and evaluating new models of care (e.g. task shifting to assistants), and developing guidelines.

To attract upcoming physicians to general practice he recommended innovative teaching concepts, a longitudinal curriculum beginning with matriculation, less ‘badmouthing’ from colleagues, and community-based clerkships. In this context, he referred to an ongoing political debate in Germany on a mandatory three-month period in general practice during the last study year as well as mandatory final exams. Regarding the five-year vocational training after graduation, he emphasized the need for a well-structured and centrally organized pathway led by academic departments of general practice. Due to the success of Szecsenyi’s project ‘Verbundweiterbildung plus’ in Baden-Württemberg [6], in Germany the establishment of so-called ‘general practice competency centres’ on the level of the federal states is discussed.

Regarding the improvement of the patient care, Szecsenyi pointed out that there is still a lack of sound data and studies evaluating the effects of the special disease management programmes for patients with chronic diseases, which have been embedded in the German healthcare system in 2002 by federal law. As important concepts that might help to improve the care for patients with chronic diseases shortly, he highlighted the potential of telemedicine and regionally integrated medical care networks. Looking further ahead, the fields of ambient assisted living devices, nursing robots, and linked information systems in healthcare were identified as promising.

The keynote ended with a humorous reference to Star Trek and the comforting knowledge that even on a high-tech starship exploring strange new worlds a GP will be needed.

## References

[1] KochK, GehrmannU, SawickiPT. Primärärztliche Versorgung in Deutschland im internationalen Vergleich. Ergebnisse einer strukturvalidierten Ärztebefragung [German Primary Care in International Comparison: Results of a Survey of Doctors]. Dtsch Arztebl. 2007;104:A2584–A2591. German.

[2] KopetschT. Dem deutschen Gesundheitswesen gehen die Ärzte aus! Studie zur Altersstruktur- und Arztzahlentwicklung [The German health care system is running out of physicians! Study on the development of age structure and number of physicians]. 5th ed Berlin: Kassenärztliche Bundesvereinigung; 2010. German.

[3] NowossadeckE. Demografische Alterung und Folgen für das Gesundheitswesen [Demographic ageing and consequences fort he health care system]. Robert Koch-Institut Berlin; 2012 GBE kompakt, 3(2). Available from: http://www.rki.de/DE/Content/Gesundheitsmonitoring/Gesundheitsberichterstattung/GBEDownloadsK/2012_2_Demografischer_Wandel_Alterung.pdf?__blob=publicationFile

[4] Sachverständigenrat zur Begutachtung der Entwicklung im Gesundheitswesen. Bedarfsgerechte Versorgung – Perspektiven für ländliche Regionen und ausgewählte Leistungsbereiche (Gutachten 2014). Berlin; 2014 Available from: http://www.svr-gesundheit.de/fileadmin/user_upload/Gutachten/2014/SVR-Gutachten_2014_Langfassung.pdf

[5] SchneiderA, GroßmannN, LindeK; DFG Network Clincial Trials in General Practice The development of general practice as an academic discipline in Germany – an analysis of research output between 2000 and 2010. BMC Fam Pract. 2012;13:58.2270247610.1186/1471-2296-13-58PMC3489844

[6] SteinhäuserJ, RoosM, HabererK, et al Bericht aus der Praxis: Das Programm Verbundweiterbildung plus des Kompetenzzentrums Allgemeinmedizin Baden-Württemberg-Entwicklung, Umsetzung und Perspektiven. Zeitschrift für Evidenz, Fortbildung und Qualität im Gesundheitswesen. 2011;105:105–109. German.10.1016/j.zefq.2011.02.00221496778

# Abstract: Chronic stress among (German) general practitioners and practice assistants [Poster]

**DOI:** 10.1080/13814788.2017.1300653

**Published:** 2017-05-11

**Authors:** Anika Thielmann, Anja Viehmann, Birgitta Weltermann

**Affiliations:** ^a^ Institute of General Medicine, University Duisburg-Essen Essen Germany

## Abstract

**Background:** Although many general practitioners (GPs) as well as practice assistants (PAs) complain about stress, no studies addressed chronic stress in both professional groups simultaneously.

**Research question:** What is the distribution of chronic stress in GP practice teams and is there a difference between single and group practices?

**Methods:** This cross-sectional study in 181 general practices measured chronic stress (strain due to stress for ≥3 months) with the validated psychometric 12-item screening-scale TICS-SSCS. Using the sum score (0–46) and the respective 75th percentiles as cut-offs (PAs > 23, female GPs > 23, male GPs > 19), each participant was categorized into low or high chronic stress. The proportions of participants with high stress were determined for the total population and separately for both professional groups. The intra-cluster correlation (ICC) was calculated for the TICS-SSC sum score.

**Results:** Data of 216 GPs (34% females) and 549 PAs (99% females) in 136 practices were analysed. The practice assistant/physician ratio was 2.8 (SD: 1.3) in single practices (54 of 136; 40%) and 2.4 (SD: 1.5) in group practices (82 of 136; 60%). Overall, 32% of single practice GPs and 40% of the GPs from group practices had high chronic stress; 43% of the PAs from single practices and 53% from group practices had high chronic stress. In single GP practices with a highly stressed physician, 53% of the PAs had high stress. In group practices with ≥1 highly stressed physician, 68% of the PAs had high stress. On the practice level, 23% of the practice members had high stress. We observed an ICC of 0.39 for all PAs and of 0.51 for GPs in group practices.

**Conclusion:** Chronic stress in GPs and PAs was more prevalent in group practices compared to single practices.

# Abstract: A systematic review to identify validated tools to assess therapeutic alliance (tool assessment for therapeutic alliance STUDY)

**DOI:** 10.1080/13814788.2017.1300653

**Published:** 2017-05-11

**Authors:** Delphine Le Goff, Michele Odorico, Bernard Le Floch, Jérémy Derriennic, Etienne Melot, Sophie Lalande

**Affiliations:** ^a^ Département de Médecine Générale, Université Bretagne Occidentale Le Relecq Kerhuon France

## Abstract

**Background:** Inside communication skills, therapeutic alliance (TA) is a relevant research theme for family medicine as it influences treatment results. TA includes four dimensions: emotional relationship; patient’s ability to follow a therapeutic target; empathic understanding and involvement of the therapist; and agreement on goals and tasks of therapy.

**Research question:** Which validated tools to assess therapeutic alliance are described in the literature (according to reproducibility, reliability and ergonomics)?

**Methods:** Medline was searched following PRISMA guidelines with the key words: ‘therapeutic alliance’ AND ‘scale’ OR ‘index.’ Inclusion criteria were: IMRAD format; scale of TA in title or abstract; scale could be used in any patient–doctor relationship (exclusion of specific scales for paediatrics, oncology, etc.); article in English or French; and minimum one reproducibility criterion.

**Results:** A total of 181 articles were selected, of which 16 were included identifying six rating scales: working alliance inventory (WAI); helping alliance questionnaire (HAQ); California psychotherapy alliance scales (CALPAS); therapeutic bond scale (TBS); Vanderbilt therapeutic alliance scale (VTAS); and Kim alliance scale (KAS). These scales had only internal validity criteria.

**Conclusion:** Scales were used exclusively in psychiatry and psychotherapy, except for the KAS. Scales WAI, HAQ and VTAS were more frequently used, probably because of the existence of short forms. The feasibility of these scales was often not mentioned. The majority of articles explored the quality of the therapeutic alliance without assessing scales, themselves. Identifying the best-validated and more adapted scale for longitudinal monitoring of TA will be the next task.

# Abstract: GP training for areas of deprivation and with marginalized groups: Does it make a difference?

**DOI:** 10.1080/13814788.2017.1300653

**Published:** 2017-05-11

**Authors:** Fiona O’Reilly, A. O’Carroll

**Affiliations:** ^a^ Partnership for Health Equity, Irish College of General Practice Dublin Ireland

## Abstract

**Background:** A 2001 UK study found non-deprived areas had almost twice the amount of applicants for GP posts as deprived areas which resulted in difficulties filling 68% of urban deprived posts compared to 29% rural posts. It is also known that marginalized groups have the worst health indices and have difficult accessing primary care. The North Dublin City GP training programme in Ireland was set up in 2009 specifically to train GPs to work in areas of deprivation and with marginalized groups.

**Research question:** How is the training programme meeting its aim to develop GPs with the desire and capacity to work in areas of deprivation and what impact is this having?

**Methods:** A mixed methods descriptive study has been implemented to capture GP trainees’ reflections and changes to attitudes as they progress through this innovative training programme. Focus group, reflective journals and pre and post-attitudinal surveys were examined. The structured programmed was reviewed and described. Follow-up of graduates was conducted. Video feedback from GP trainees was also captured.

**Results:** Preliminary data demonstrates that GP registrars actively seek to include deprived and marginal groups in their practice post graduating. Data demonstrates changes in attitudes. These posts involve GP registrars working one day a week for four months in homeless primary care services; another day in prison and ethnic minority primary care services; and another in drug treatment services. The educational theory for these posts is based on the contact hypothesis which proposes that contact breaks down stereotypes. Videos of registrar feedback on these posts demonstrate this.

**Conclusion:** This innovative programme has produced results that suggest that, through training, GPs can be better equipped with capacity and desire to work in areas of disadvantage and marginalized groups. Expansion of this model of training is recommended.

# Abstract: Perspectives on developing family medicine training in the West Bank

**DOI:** 10.1080/13814788.2017.1300653

**Published:** 2017-05-11

**Authors:** Hina J. Shahid, Mohammed Rabai, David Jewell

**Affiliations:** ^a^ Foundation for the International Development of Family Medicine in Palestine London U K

## Abstract

**Background:** After two initial attempts at establishing family medicine training in the West Bank, the future of the programme has once again been called into question. We aim to analyse barriers to its implementation and how to overcome these.

**Research question:** What are the key informants’ perspectives on the development of the family medicine training programme in the West Bank?

**Methods:** (1) Study design: a mixture of semi-structured interviews with key informants and field observations. Three weeks fieldwork with follow-up emails and phone calls with collaborators. Documentary analysis of internal correspondence and reports. (2) Study setting: West Bank cities (Hebron, Ramallah, Nablus) and surrounding towns and villages. (3) Participants: family medicine specialists recently qualified; representatives from the Ministry of Health and Palestine Medical Council; faculty members from An-Najah University’s Department of Community Health and Family Medicine; and project workers from UK-based NGOs. (4) Outcomes: qualitative analysis using the SCOT framework of strengths, challenges, opportunities and threats to analyse the data.

**Results:** The Ministry of Health has identified family medicine as a national priority for development. Challenges include physical, technical and human resources; academic opportunities; supervision; strategic planning; and complex sociopolitical circumstances. Key stakeholders express optimism that expanding FM is essential to achieve universal health coverage in Palestine and it is attainable through a coordinated multi-sectoral effort and supportive international partners.

**Conclusion:** Family medicine is seen positively in the West Bank with several strengths and opportunities. Stakeholders need to collaborate to address the remaining challenges. The study has included a wide range of perspectives and multiple data sources. We hope it will be a useful tool to assess current progress and guide future planning.

# Abstract: Joint action health workforce—Belgian pilot project: Horizon scanning in general practice

**DOI:** 10.1080/13814788.2017.1300653

**Published:** 2017-05-11

**Authors:** Lieve Peremans, Jessica Fraeyman, Jean Macq, Maguelone Vignes, Roy Remmen

**Affiliations:** ^a^ University of Antwerp, Primary and Interdisciplinary Care Antwerpen Belgium

## Abstract

**Background:** Current health workforce planning is more than a demographic exercise on replacing retiring GPs but needs to assess the impact of other factors within a changing society. Belgium uses a mathematical model for the calculation of the health workforce planning.

**Research question**: How can qualitative research methods be used for the development of new parameters for this model.

**Methods:** This study is part of the EU-project ‘Joint Action on Health Workforce Planning and Forecasting’. We interviewed a purposive sample of 16 critical cases, from target organisations identified by the Federal Public Health Service. The interview guide was based on an international framework used for horizon scanning. For the Delphi, we selected two factors: the evolution in GP demand and future activity rate. The Delphi was completed by 27 experts in the second round.

**Results:** There were concerns about the current role of GPs mostly working in single-handed practices. Those GPs are unable to deliver integrated care needed for people with co-morbid conditions. In the Delphi, experts have different opinions on the needs in both parts of the country but overall, the median change for the group above 80 years is expected to be up to 25%. There is an oversupply of candidate physicians, but all the experts pointed out the imminent retirement GPs. Indeed, 74.16% of active Belgian GPs were more than 45 years old in 2012. New GPs are predominantly female, with a strong reduction in activity rate. Task delegation and the introduction of new professions such as nurse practitioners might provide a solution but a change in the legal framework is needed.

**Conclusion:** This qualitative research raises new ideas, but they are not always easy to translate to a mathematical model. Experts and policymakers must develop a vision for healthcare organization before a more elaborate model can be used.

# Abstract: Influenza vaccination during pregnancy: Prevalence and barriers to uptake?

**DOI:** 10.1080/13814788.2017.1300653

**Published:** 2017-05-11

**Authors:** T. Barrett, E. McEntee, Aisling O’Shea, B. Cleary, R. Drew, Claire Collins, Fiona OReilly, Aisling O’Shea

**Affiliations:** ^a^ Partnership for Health Equity, Irish College of General Practice Dublin Ireland

## Abstract

**Background:** Flu vaccine is recommended during pregnancy. MMBRACE UK’s report found that one in eleven maternal deaths was caused by influenza. In spite of recommendations, the little data that exists on the uptake in Ireland suggests uptake is less than optimal. There remains ambiguity about where responsibility for vaccination lies. This study aims to address such ambiguity by informing future policies.

**Research question**: What is the prevalence of and barriers to uptake of influenza vaccine in pregnancy?

**Methods:** Knowledge, attitudes and practices of women post-delivery were assessed in a maternity hospital in Dublin, Ireland. This was complemented by in-depth interviews with a small group of women within six weeks of birth to allow further exploration of their views and experience. The third component involved an online survey targeted at GPs, hospital doctors and pharmacists.

**Results:** A total of 150 women participated within 48 h post-delivery, 1181 health providers completed an online survey and 12 women participated in qualitative in-depth interviews. Preliminary findings suggest that uptake of influenza vaccine has increased with 88 (61%) of mothers post-delivery reporting vaccination. Among healthcare providers, knowledge was variable with 70% providing correct answers on knowledge of risks relating to influenza during pregnancy and 50% correct answers of knowledge relating to the benefits of vaccination. Three-quarters (75%) of GPs had been vaccinated themselves compared to 58% of pharmacists (chi^2^ 36.6, *P* < 0.01). Qualitative interviews with women highlight the positive potential of healthcare provider recommendation and that this is not always utilized.

**Conclusion:** Results indicate an increase in the uptake and acceptability of influenza vaccine during pregnancy. The positive influence by healthcare workers including hospital doctors, whose influence is likely to have an impact, requires further support through a clear national policy statement.

# Abstract: A decision aid to use within pre-screening-counselling about the PSA-test: Practical support or time-consuming impeder?

**DOI:** 10.1080/13814788.2017.1300653

**Published:** 2017-05-11

**Authors:** Kathrin Schloessler, Charles Christian Adarkwah, Alexandra Simbrich, Matthias Borowski, Norbert Donner-Banzhoff, Axel Semjonow, Hans Werner Hense, Peter Maisel, Katrin Kuss

**Affiliations:** ^a^ Philipps University Marburg, Family Medicine Marburg Germany

## Abstract

**Background:** The value of PSA-screening for prostate cancer is a matter of current debate. Patients individually have to weigh benefits and harms. However, this is not a simple task for patients or their consultant physician as some studies found a lack of shared decision making (SDM) and evidence-based information in pre-screening discussions.

**Research question:** Is a transactional decision aid (DA) on PSA-screening a useful and viable innovation to support physicians in pre-screening counselling?

**Methods:** According to the International Patient Decision Aid Standards, we developed a first draft of a DA based on best available evidence. This version was field tested in two qualitative studies with GPs and urologists, respectively. Both groups counselled men aged 55–69 (*n* = 32). All participants interviewed using were semi-structured interviews; interviews were transcribed verbatim and analysed qualitatively combining deductive categories with emerging themes.

A pilot study assessed study procedures and implementation. Here GPs were randomized to either apply or not apply the DA (‘as usual’). Participating men received questionnaires regarding their current decision; decision quality and process (SDM). The GPs were interviewed about their experiences in daily practice.

**Results:** The qualitative study revealed feedback that led to substantial changes in the DA. Overall, the DA was seen as a helpful tool for pre-screening discussions. While physicians had concerns about the time required, their reported counselling time had a wide range. Interestingly, some physicians changed their own attitude about PSA-screening and learned new information by using the DA. In our pilot study, aspects of SDM were found in both the intervention and control groups. However, our analysis remains descriptive due to a small sample size (*n* = 178).

**Conclusion:** The use of a DA on PSA-screening during consultation was feasible and might contribute to neutral counselling based on best available evidence.

# Abstract: Boon or bane? Arriba^®^PSA: A new decision aid to support PSA-screening counselling

**DOI:** 10.1080/13814788.2017.1300653

**Published:** 2017-05-11

**Authors:** Charles Christian Adarkwah, Katrin Kuss, Norbert Donner-Banzhoff, Axel Semjonow, Hans-Werner Hense, Alexandra Simbrich

**Affiliations:** ^a^ Department of General Practice/Family Medicine, University of Marburg Marburg Germany

## Abstract

**Background:** The value of PSA-screening for prostate cancer is a matter of current debate. Nevertheless, experts agree on the fact that patients need to be informed about risks, benefits and consequences before testing. Evidence-based decision aids (DAs) are able to support the counselling process and increase decision quality. We developed the DA arriba^®^PSA, which has already been tested in qualitative pre-studies.

**Research question**: Is counselling with the DA arriba^®^PSA superior compared to counselling without a DA concerning the decisional conflict and the degree of information?

**Methods:** A cluster-randomized, controlled trial was performed, involving 28 general practitioners who consecutively recruited 169 patients. GPs were randomized to either apply (*n = 7*5) or not apply the DA (*n* = 94; ‘as usual’). Participants filled in questionnaires regarding their current decision, decision quality and process. Main outcomes are the degree of information and the decisional conflict, measured by the decisional conflict scale (DCS).

**Results:** Arriba^®^PSA is associated with a higher degree of information. The scores in the DCS are low in both groups and there are no significant differences to be found.

**Conclusion:** More information might allow for a better decision regarding the decision for or against PSA screening. The DA is applicable in daily practice and might contribute to neutral counselling based on the best available evidence.

# Abstract: Effects of lowering treatment threshold for statin therapy in a German population-based sample

**DOI:** 10.1080/13814788.2017.1300653

**Published:** 2017-05-11

**Authors:** Aniela Angelow, Gesine Weckmann, Jean-François Chenot

**Affiliations:** ^a^ Department of General Practice, Institute for Community Medicine, University Medicine Greifswald Greifswald Germany

## Abstract

**Background:** Statin therapy is recommended for primary prevention of cardiovascular disease (CVD) in patients with high CVD risk and all patients with CVD. Recent guidelines lowered the treatment threshold to moderate risk. There are few data on the impact of the implementation of these recommendations on clinical practice in Germany.

**Research question:** What is the estimated proportion of patients with guideline-concordant statin treatment? What is the estimated effect of lowering the statin treatment threshold from high to moderate CVD risk on statin treatment rates?

**Methods:** We analysed data of the population based cohort Study of Health in Pomerania covering a 10-year period. CVD risk factors and statin therapy were defined based on structured interview data, clinical measurements and a medication review. CVD risk was estimated using SCORE-Germany. Subjects with diabetes or prior history of myocardial infarction were classified as high CVD risk (≥5%). Only descriptive analyses were performed.

**Results:** Overall 1365 subjects aged 40 to 69 years (46% male, mean age 53.1 years, SD: 7.6 years) were included. Based on a high CVD risk, 3%, 24% and 58% of subjects aged 40–49, 50–59 and 60–69 years should receive statin treatment. Statin treatment rates in subjects at high CVD risk aged 40–49, 50–59 and 60–69 years were 36%, 25% and 29%. If statin treatment was extended to subjects with moderate CVD risk, 10%, 56% and 93% would be eligible (overall proportion: 49%).

**Conclusion:** Statin therapy is underused in patients with high CVD risk. However, if treatment is extended to subjects at moderate CVD risk, approximately half of the population aged 40–69 years would be eligible for statin treatment. Given the known overestimation of CVD risk, a more individualized threshold for statin therapy could prevent over-medicalization.

# Abstract: Implications of referral criteria for chronic kidney disease—analysis of a population based sample

**DOI:** 10.1080/13814788.2017.1300653

**Published:** 2017-05-11

**Authors:** Gesine Friederike Weckmann, Jean François Chenot, S. Stracke, A. Haase, S. Kiel, J. Spallek, A. Angelow

**Affiliations:** ^a^ Department of General Practice and Family Medicine, Institute for Community Medicine Greifswald Germany

## Abstract

**Background:** Chronic kidney disease (CKD) has an age-dependent prevalence of 10% in adults. The majority of CKD patients are only seen in general practice. German societies for nephrology and internal medicine recommend specialist referral for all subjects with estimated glomerular filtration rate (eGFR) < 45 or eGFR 45–59 ml/min/1.73 m^2^ with albuminuria, haematuria, hypertension or anaemia.

**Research question:** What proportion of the general population meets the recommended referral criteria for CKD?

**Methods:** Data from the population based cohort Study of Health in Pomerania (SHIP-2) were analysed to estimate the proportion of subjects who meet the proposed referral criteria.

**Results:** Data of 2328 subjects from SHIP-2 (53% female; age mean = 57 years, SD: 14) were analysed. Overall, 3% of subjects had eGFR <45 ml/min/1.73 m^2^ and 6% had an eGFR between 45 and 59 ml/min/1.73 m^2^. Of those, 8% had albuminuria, 42% haematuria, 67% hypertension and 45% anaemia. Referral criteria were met by 0% of subjects aged 30–49 and 1%, 8%, 20% and 41% of those aged 50–59, 60–69, 70–79 and ≥80 years (overall rate 8%).

**Conclusion:** Adherence to the proposed referral criteria would increase the number of referrals to nephrology substantially, particularly of the elderly. Given a lack of a specific nephrological therapy, the benefit of increasing referrals beyond the capacity of nephrological workforce seems doubtful. Referral criteria for common medical disorders in general practice should be evaluated rigorously.

# Abstract: Enhancing primary healthcare (PHC) services for refugees and migrants reaching EU countries.The EUR-HUMAN project

**DOI:** 10.1080/13814788.2017.1300653

**Published:** 2017-05-11

**Authors:** Mechili Enkeleint-Aggelos, Agapi Angelaki, Valeria Chatzea, Dimitra Sifaki-Pistolla, Danica Rotar-Pavlic, Kathryn Hoffman

**Affiliations:** ^a^ Clinic of Family and Social Medicine, School of Medicine, University of Crete Heraklion Greece

## Abstract

**Background:** Europe is facing a major issue with refugees and migrants. Although several studies have reported on the impact of the refugee crisis on health, an integrated attempt to deliver primary healthcare (PHC) services based on refugees’ healthcare is lacking. The EUR-HUMAN project aims to enhance the capacity, knowledge and expertise of European countries who accept refugees/migrants in addressing their health needs.

**Research question:** How can general practitioners (GPs) provide person-centred, continuous, coordinated and comprehensive PHC services to refugees/migrants?

**Methods:** The EUR-HUMAN project utilized evidence-based methodological and theoretical approaches including the participatory and learning action (PLA), the chronic care model and the ‘mental health psychosocial support’ model. Data were collected from multiple sources including: PLA focus groups with refugees, literature review jointly with cross-national survey and expert interviews, and a consensus expert meeting that summarized and synthesized the evidence.

**Results:** According to the PLA method, 98 refugees participated in 43 sessions in seven countries (Greece, Hungary, Italy, Austria, the Netherlands, Slovenia, and Croatia). The main health problems mentioned by the participants were related to conflicts and the journey they had to undertake. The results revealed important barriers in accessing PHC services such as time pressure, linguistic and cultural differences, and lack of continuity of care. The systematic literature review revealed obstacles linked to: healthcare interventions and measures of evaluation, professional–patient interaction, incentives and resources, local capacity for organizational change, and social, political, and legal factors. Ten steps to initial psychosocial aid were identified. The consortium translated all these findings into best practice guidelines, tools and services aiming to enhance capacity building and knowledge of first line healthcare professionals and GPs.

**Conclusion:** The project promotes comprehensive, person-centred and integrated health need assessment based on refugees’ preferences and the lessons learned. The feasibility and acceptability of the planned interventions will be assessed in pilot interventions.

# Abstract: Volunteering in Serbia: Medical and psychosocial relief operation for the Middle-East refugees crossing Europe [Poster]

**DOI:** 10.1080/13814788.2017.1300653

**Published:** 2017-05-11

**Authors:** Sarah Sigalat, May Kyttany, Khaled Karkabi

**Affiliations:** ^a^ Family Medicine, Rappaport Faculty of Medicine, Technion, Haifa, Clalit Health Services Haifa Israel

## Abstract

**Background:** Thousands of refugees from Syria, Iraq and Afghanistan were arriving daily at the transit camp of Presevo, Serbia. They did their journey by boats from Turkey to Greece, then by trains to Macedonian border, walking six kilometres to Presevo, and onwards to Croatia.

**Research question:** To evaluate medical assistance to refugees from the Middle East during the volunteering period.

**Methods:** NATAN, which is an international humanitarian aid organization, has been sending teams including physicians, nurses, social workers and logisticians, for two to four weeks. The clinic, which was an equipped container, operating on a 24/7 basis, was established in Presevo Camp. The work included registration of the refugees’ name, age, gender, country of origin, diagnosis, and treatment. Tests performed included: blood pressure, temperature, glucose and urine tests.

**Results:** For six months, beginning 9 November 2015 and ending on 27 April 2016, 46 Israeli volunteers, Jews and Arabs, offered their skills and time in a very challenging situation. More than 50% of professionals were Arabic-speaking and had an understanding of the refugees’ culture. Overall, 3600 refugees, 40% children, received direct primary care by 12 doctors. During my 10 days volunteering, 385 refugees were treated by my colleagues and me. Most complains were URI, pneumonia, hypothermia, back and leg pain, headache and insomnia. We treated infected and shooting wounds, frost bite, bandaging, injections, intravenous fluid and antibiotic therapy.

**Conclusion:** Bio-psycho-social care gave immediate support to people in need. The refugees were relieved to receive healthcare delivered by professionals speaking their language. It was rewarding to observe Israeli, Jewish and Arab doctors, working side-by-side caring for refugees from Arab countries. Medical volunteering is a great challenge and unique experience for family doctors, working together with other organizations. We felt that we were in the middle of a historical nations’ movement to Europe.

# Abstract: Electronic reminders to facilitate chronic care: A mixed-methods study in primary care [Poster]

**DOI:** 10.1080/13814788.2017.1300653

**Published:** 2017-05-11

**Authors:** Christine Kersting, Birgitta Weltermann

**Affiliations:** ^a^ University of Duisburg-Essen, Institute for General Medicine Essen Germany

## Abstract

**Background:** Longitudinal, patient-centred chronic care is a challenge for primary care physicians. German practices facilitate this challenge by generating self-designed reminders.

**Research question:** This study aimed to identify details on reminders used in primary care: (1) the contents of care addressed, (2) the reminders’ actual design, (3) their functionalities, and (4) their routine use.

**Methods:** This mixed-methods study combined a cross-sectional survey among 185 primary care practices from a German university network, and structured observations of reminder utilization in six practices. Descriptive statistics were performed for survey data; practice observations were analysed by grouping the different reminder systems about their designs and functions.

**Results:** A total of 73 of 185 practices completed the survey (40%): 98.6% use reminders in the (electronic) health records (HR/EHR). Frequent care contents addressed are allergies/risks (96%), preventive measures/check-up (93%), participation in disease management programme (88%), chronic diseases (75%), and vaccinations (68%). Practice observations showed different, mainly self-configured reminders with one practice using paper-based reminders. Information was presented (1) continuously in a separate field, (2) scattered through the EHR, and/or (3) in pop-up windows. The design of electronic reminders varied: (1) coloured fields with short text, (2) words in coloured letters, (3) abbreviations, (4) symbols, (5) EHR-embedded information buttons marked by symbols, e.g., a light-bulb, and (6) pre-structured fields from software add-ons. Five practices develop reminders ‘as needed’; one practice applied a comprehensive, EHR-predefined reminder system. Practices had reminders for a mean of 13.3 aspects of care (range: 9–21; standard deviation (SD): 4.3) from 26 aspects outlined in a clinical vignette. Time to retrieve the information for the standardized clinical vignette took from 20 to 35 minutes (mean: 27.5; SD: 6.1).

**Conclusion:** Most practices use visual reminders for selected aspects of chronic care, yet data-based, sophisticated solutions are required to improve care.

# Abstract: Repercussions of the use of the ZARIT scale on the care of patients with Alzheimer’s disease and their family caregivers in primary care: A systematic review of the literature [Poster]

**DOI:** 10.1080/13814788.2017.1300653

**Published:** 2017-05-11

**Authors:** Julie Grosse, Clarisse Dibao-Dina, J. Robert

**Affiliations:** ^a^ DUMG Tours, Faculté de médecine de Tours Amboise France

## Abstract

**Background:** The role of family caregivers of patients with Alzheimer’s disease has many physical and psychological consequences that can increase the caregiver’s burden. French guidelines recommend using the ZARIT scale to evaluate this burden. However, the relevance of the ZARIT in practice is unclear, particularly regarding consequences on the dyad’s care (patient and caregiver).

**Research question:** What are the repercussions of the use of the ZARIT scale on the care of patients with Alzheimer’s disease and their family caregivers in general practice?

**Methods:** Systematic review of the literature on five international (Medline, Cochrane, Psycinfo, Francis, Science direct) and three French databases with a MESH search equation (Cognition disorders [MeSH Terms] OR Alzheimer disease [MeSH Terms]) AND zarit AND burden AND caregivers [MeSH Terms]). The electronic search was performed in June 2014. Selection criteria were articles on family caregivers, setting in primary care and with a performed ZARIT scale. Articles on ZARIT determinants were excluded. Data was collected with a tested data extraction form and concerned characteristics of studies and population (patients and caregivers), ZARIT scale performances and its consequences.

**Results:** Of 285 eligible articles, 12 were included. All studies were observational and two studies were exclusively in primary care. Patients were 77 years old on average and caregivers 60 years old. An elevated ZARIT score was associated with an increased risk of hospitalization, institutionalization and abuse of the patient with Alzheimer’s disease. It was also associated with a risk of psychopathological deterioration for the caregiver. A persistently elevated level of burden was a predictive factor of depression in caregivers. There was no study evaluating an intervention based on the ZARIT scale to improve the dyad’s care.

**Conclusion:** Studies are needed with interventions based on the ZARIT scale to evaluate its relevance in general practice.

# Abstract: Occurrence and associated factors of malnutrition in community-dwelling older adults [Poster]

**DOI:** 10.1080/13814788.2017.1300653

**Published:** 2017-05-11

**Authors:** Tine De Burghgraeve, Carine van den Broeke, Laura Deckx, Frank Buntinx, Marjan van den Akker

**Affiliations:** ^a^ KU Leuven, Public Health and Primary Care Leuven Belgium

## Abstract

**Background:** In older adults, nutritional health is essential for good quality of life and living independently at home. Especially in cancer patients, malnutrition is common and known to complicate treatment.

**Research question:** This study aims to evaluate the nutritional status and its associated factors in community-dwelling older adults with and without cancer.

**Methods:** This study was embedded in the Klimop study, an observational cohort study which included older people with and without cancer (≥70 years). Cancer patients included patients with a new diagnosis of breast, lung, prostate, or colorectal cancer. Data collection included measures of nutritional status, quality of life, depression, fatigue, distress and functional status. We used multivariate logistic regression analysis to assess the association between personal characteristics and malnutrition.

**Results:** Data was available for 657 people; 383 people without cancer and 274 with a cancer diagnosis. Overall, malnutrition was detected in 245 (37.5%) people; in cancer patients, this was 66.1%. Multivariate analysis showed that having cancer (OR: 13.36, 95%CI: 8.40–21.25), being female (OR: 2.38, 95%CI: 1.49–3.70), having depression (OR: 12.46, 95%CI: 5.54–28.03), distress (OR: 2.32, 95%CI: 1.37–3.91) and impaired instrumental activities of daily living (IADL) (OR: 2.63, 95%CI: 1.64–4.21) were associated with a higher risk of malnutrition.

**Conclusion:** The prevalence of malnutrition in community-dwelling older people is high, particularly in patients with cancer. Benchmarking and routine screening of older patients may be helpful strategies to increase awareness of (risk of) malnutrition among professionals.

# Abstract: Depending decisioning—Danish and Swedish primary care physicians’ decision making and diagnostic reasoning in cancer work up—a grounded theory study

**DOI:** 10.1080/13814788.2017.1300653

**Published:** 2017-05-11

**Authors:** Hans Thulesius, Ulrika Sandén, Berit Skødeberg Toftegaard, Peter Vedsted

**Affiliations:** ^a^ Family Medicine, Lund University Växjö Sweden

## Abstract

**Background:** Diagnostic workup of cancer is complex in primary care where most symptomatic cancers are suspected and eventually detected. Conceptual understanding of what goes on when physicians are engaged in workup of cancer is still weak.

**Research question:** What happens when primary care physicians (PCP) see patients having symptoms that may indicate cancer?

**Methods:** Grounded theory analysis of written commentaries from Danish and Swedish PCPs responding to an internet survey in 2012. The survey’s clinical vignettes assessing management of diagnosis of lung, colorectal or ovarian cancer were analysed. A total of 237 out of 255 (93%) Danish and 165 out of 198 (83%) Swedish PCPs wrote open-ended comments in their words to one or more of the items in the survey. We did classic grounded theory analysis: open coding, theoretical memoing, selective coding, theoretical sorting and writing up.

**Results:** Cancer diagnosis can be explained by PCPs engaging in a pattern of behaviour we call Depending or Conditioned Decisioning which has a dual meaning referring to: (i) contextual conditions—requirements, steps and routines—in the cancer workup procedure as well as (ii) biomedical conditions—the decisioning contingent on the type of condition(s) the patient is suffering from. Depending Decisioning is sequential and stratified. It starts with recognizing signs and symptoms followed by investigational strategizing adapted, deliberated and reflected upon contingent on contextual and biomedical conditions. Depending Decisioning involves both sequential planning of tests and investigations and execution of tests. The physician elaborates a heuristic plan for future investigations and tests in sequence based on the results of present tests. The plan includes a gauging of both the patient’s biomedical condition and consultation contextual conditions.

**Conclusion:** We present Depending Decisioning as a conceptual understanding of what goes on during PCPs’ decision making and workup of cancer.

# Abstract: Drug safety of patients with rheumatoid disorders treated with methotrexate

**DOI:** 10.1080/13814788.2017.1300653

**Published:** 2017-05-11

**Authors:** Jean-François Chenot, Aniela Angelow, Armin Mainz, Jochen Walker

**Affiliations:** ^a^ General Practice, University Medicine Greifswald Greifswald Germany

## Abstract

**Background:** Methotrexate (MTX) is an immunosuppressant for long-term treatment of rheumatoid disorders. Potentially live threatening serious adverse events (SAE) are nephrotoxicity, hepatotoxicity and blood dyscrasias. Therefore manufacturers and medical societies recommend periodic monitoring examinations and folic acid supplementation.

**Research question:** How frequent are serious adverse events of MTX and do patients receive the recommended monitoring examinations and prescriptions for folic acid?

**Methods:** This is a retrospective observational study of anonymized claims data from seven million subjects covered by statutory health insurance. We identified 40 087 patients who received prescriptions for MTX from 2009 to 2013. A total of 12 451 patients newly started on MTX remained after restriction to adults with rheumatoid disorders (ICD M05–18) without MTX prescription for 12 months before the index prescription. They were followed for up to 90 days beyond the reach of the last prescription. Mean follow-up time was 476 days. During this period we analysed the billing codes for laboratory examinations and coding of SAE.

**Results:** Acute kidney failure was observed in 3.48, liver failure in 0.68 and aplastic anaemia in 0.93 patients per 1000 person years. The proportion of patients receiving all recommended controls was 46% for CBC, 42% for GGT and ALAT, 43% creatinine and 14% urine dip stick. Discrepancies were mainly observed at the beginning. Overall, 73% received prescriptions for folic acid. Most patients (83%) where seen at least once for an annual follow-up by the rheumatologist.

**Conclusion:** The recommended monitoring examinations for patients on MTX and folic acid supplementation are only partly followed. The omission of recommended monitoring examinations might pose a risk for patients and a forensic risk for doctors, although the effectiveness of this measure in preventing SAE is unproven.

# Abstract: Do multi-children women have higher risk of osteopenia and osteoporosis? A study from Western Ukraine

**DOI:** 10.1080/13814788.2017.1300653

**Published:** 2017-05-11

**Authors:** Pavlo Kolesnyk, Ivan Kutchak, A. Dolgikh

**Affiliations:** ^a^ Uzhgorod National University, Family and Internal Medicine Uzhgorod Ukraine

## Abstract

**Background:** Prevalence of osteoporosis occurs in women of postmenopausal age. Densitometry is devoted providing screening in this cohort. However osteopenia can be found in fertile multi-children women.

**Research question:** Can multi-children mothers become a considered cohort for screening of osteoporosis before menopausal age?

**Methods:** A total of 270 record forms of women with more than three children including Roma ethnic group from rural western region of Ukraine were processed. Two groups of women have been selected depending on the number of pregnancies in their history. The average number of pregnancies in women in the index group was 4.36 ± 1.62. Women were divided into subgroups—fertile and menopausal. We used a questionnaire which evaluated risk factors and women’s nutrition. Bone mineral density (BDM) and T-score was examined using calcaneal quantitative ultrasound densitometer ‘Sahara Hologic.’

**Results:** Over 46% of examined multi-children women had osteopenia comparing with 35.7% of the control group. This difference was not significant among fertile women of examined groups. The frequency of osteopenia and osteoporosis depended on age (rs = –0.42; *P* > 0.05) and was significantly higher among menopausal women. A weak positive correlation was found between BDM (T-score) and number of pregnancies among examined fertile women (rs = 0.29, *P* = 0.356) and calcium content in their diet (rs = 0.109, *P* = 0.736). Prevalence of osteopenia and osteoporosis did not depend on the ethnicity of examined women. Though no significant difference in BMD of fertile women has been observed, frequency of osteopenia and osteoporosis occurred significantly higher among postmenopausal women who have had more than three pregnancies.

**Conclusion:** Though the number of pregnancies does not correlate with higher level of osteopenia and osteoporosis in fertile age, the cohort of multi-children women can be considered as a risk group of developing osteopenia in early menopausal age.

# Abstract: Multi-drug use in hypertensive patients at a family medicine out-patient clinic of a Turkish University Hospital [Poster]

**DOI:** 10.1080/13814788.2017.1300653

**Published:** 2017-05-11

**Authors:** Zehra Dagli, Canan Tuz, Filiz AK, Ayse Selda Tekiner, Ayse Gülsen, Ceyhun Peker, Mehmet Ungan

**Affiliations:** ^a^ Department of Family Medicine, Ankara University School of Medicine Ankara Turkey

## Abstract

**Background:** In Turkey, the most common diagnosis in prescriptions is primary hypertension (9.29%) and mean number of drugs per one prescription is 2.46. The study was carried out to detect the prevalence of multi-drug use in hypertensive patients and the risks of interaction and adverse reaction with the drugs prescribed for accompanying conditions.

**Research question:** What is the prevalence of multi-drug use in hypertensive patients? Do the drugs prescribed for accompanying conditions interact with antihypertensive agents? Do the concurrent drugs affect hypertensive therapy?

**Methods:** The retrospective, cross-sectional, descriptive study included all individuals who attended a family medicine outpatient clinic at a university hospital for their anti-hypertensive medications to be prescribed for the first time in 2014 and 2015. Patients with any cardiovascular, cerebrovascular, endocrinological, metabolic and nephrological conditions which could interfere with anti-hypertensive therapy were excluded. Only the agents used systemically were taken into account. Continuous and categorical variables were given as mean ± standard deviation and percentages, respectively.

**Results:** Mean age of 150 patients (41.3% male, 58.7% female) was 58.99 ± 13.66 years. Mean number of active anti-hypertensive agents prescribed was 1.74 ± 0.76 (range: 1–5). Of the patients, 42% used only one anti-hypertensive agent, 44.7% used two agents. The most frequent anti-hypertensive drugs prescribed were combinations of angiotensin receptor blockers and diuretics (30.6%), beta-blockers (24.0%), and calcium channel blockers (20.0%). Sixty-six per cent of the patients were prescribed concurrent drugs. Mean number of agents prescribed for accompanying diseases was 1.45 ± 1.57 (range: 0–8). The most common agents prescribed were non-steroidal anti-inflammatory drugs (25.3%). While 36.7% patients received drugs interacting with anti-hypertensive agents, 23.3% received drugs with adverse effect of hypertension.

**Conclusion:** Anti-hypertensive drugs prescribed seemed to be accordant with current guidelines. As two-thirds of the patients used other drugs along with anti-hypertensive agents, rational drug use should be considered while prescribing routine drugs.

# Abstract: Diabetic retinopathy in Catalonia: Association with cardiovascular outcomes and chronic kidney disease [Poster]

**DOI:** 10.1080/13814788.2017.1300653

**Published:** 2017-05-11

**Authors:** Antonio Rodriguez-Poncelas, Sonia Miravet, Joan Barrot, Flora Lopez-Simarro

**Affiliations:** ^a^ Universitat Autonoma Barcelona, Medicine Barcelona Spain

## Abstract

**Background:** The presence of diabetic retinopathy (DR) is related to cardiovascular risk factors and consequently a greater incidence of cardiovascular disease. Chronic kidney disease (CKD) and DR reflected similar lesions in the glomerular and retinal vessels.

**Research question:** To determine cardiovascular outcomes and CKD associated with DR in type 2 diabetes (T2DM) in primary health care.

**Methods:** Cross-sectional population study of T2DM patients (*n* = 329 410) with RP were selected (*n* = 108 723; 33%). DR was classified as normal, non-vision threatening retinopathy (non-VTDR) and vision threatening retinopathy (VTDR). Cardiovascular disease (CVD) was defined as stroke or coronary heart disease (CHD). CKD was defined as estimated glomerular filtration ratio (eGFR) of <60 ml/min/1.73 m^2^ and/or urine albumin to creatinine ratio (UACR) > 30 mg/g. Clinical Information was obtained from the SIDIAP database (system for research and development in primary care).

**Results:** Of the patients analysed (55% men), the mean age was 66.9 years (SD: 11). Mean duration of T2DM was 7.8 years (SD: 5.1). Patients with any DR had higher HbA1c mean value (7.8% versus 7.2%) and prevalence of hypertension (85.2% versus 79.3%), glomerular renal filtration <60 ml/min (MDRD) (26.1% versus 18.9%) and albumin:creatinine ratio >30 mg/g (27.4% versus 15.5%) than the rest. In the multivariate analysis of CVD, the effect of DR was non-VTDR (OR: 1.24, 95%CI: 1.15–1.34) and VTDR (OR: 1.32, 95%CI: 1.09–1.60); CKD was associated with a higher rate of DR (OR: 1.5, 95%CI: 1.4–1.7). DR prevalence rose with the increase of UACR levels. This association was significant from UACR values =10 mg/g, and increased considerably with UACR value >300 mg/g (OR: 2.0, 95%CI: 1.6–2.5). This association was lower in patients with eGFR levels 44–30 ml/min/1.73 m^2^ (OR: 1.3, 95%CI: 1.1–1.6).

**Conclusion:** The presence of RD was related to CVD event and CKD. High UACR and/or low eGFR, appear to be associated with DR in the T2DM population.

# Abstract: Smoking and non-smoking diabetic patients, are they different? [Poster]

**DOI:** 10.1080/13814788.2017.1300653

**Published:** 2017-05-11

**Authors:** Carlos Martin Cantera, Inka Miñambres, Susana Fontana, Victoria Feijoo, Elisa Puigdomenech, Lorena Soto

**Affiliations:** ^a^ Institut Català de la Salut, Primary Care Barcelona Spain

## Abstract

**Background:** Smoking in patients with type 2 diabetes (T2D) is related to unfavourable effects. Information concerning the characteristics of smoking patients with T2D is scarce and may help to implement strategies for smoking cessation.

**Research question:** Do clinical characteristics differ among non-smoking and smoking T2D patients?

**Methods:** From a database that includes 1 047 008 individuals aged 15 years or older who attended at primary care centres in Barcelona (Spain), we selected and extracted data of T2D patients from 1 January to 31 December 2014. Demographic and clinical characteristics where analysed. Descriptive and bivariate analysis were undertaken to compare smoking and non-smoking patients.

**Results:** A total of 73 490 T2D patients were included, mean age: 70.4 ± 12.3; 53.9% men. The percentage of smokers, former smokers and non-smokers was 15.6%, 10.5% and 73.3%, respectively. When compared to T2D non-smokers, smokers were predominantly men (21.8% versus 8.3% women) and younger (62.1 ± 10.9 versus 72.2 ± 11.8 years). Smokers had higher diastolic blood pressure (76.4 ± 10.2 versus 73.9 ± 9.9), higher LDL cholesterol (106.6 ± 35.0 versus 104.6 ± 36.3) and HbA1c (7.2 ± 1.4 versus 7.02 ± 1.2) and lower levels of HDL cholesterol (45.7 ± 12.8 versus 50.3 ± 19.1). Their diabetes duration was longer (6.8 ± 5.1 versus 8.4 ± 6.1 years) and presented more frequently treatment with insulin (24.9% versus 22.7%) and peripheral arterial disease (10% versus 5.6%). Ex-smokers presented more cardiovascular disease than non-smokers (46.1% versus 38.4%). Finally, smokers had a lower number of annual visits with general practitioners (7.8 ± 6.6 versus 8.9 ± 6.9) and nurses (7.6 ± 9.8 versus 8.9 ± 11.2). More anxiety diagnosis was also registered (12.1% versus 10.2%).

**Conclusion:** T2D patients who smoke are mainly men and younger than non-smokers and have worse metabolic control. Anxiety and a lower attendance at primary care centres are common.

# Abstract: European study on self-care for common colds: An analysis of self-care and potential medication interactions (COCO study)

**DOI:** 10.1080/13814788.2017.1300653

**Published:** 2017-05-11

**Authors:** Biljana Gerasimovska Kitanovska, Anika Thielmann, Krzysztof Buczkowski, Ferdinando Petrazzuoli, Enzo Pirrotta, Robert D. Hoffman, Aysegul Uludag, Kathryn Hoffmann, Slawomir Czachowski, Tamer Edirne, Tuomas Koskela, Heidrun Lingner

**Affiliations:** ^a^ University Clinical Centre, Nephrology Skopje Macedonia

## Abstract

**Background:** Self-care for common colds is frequent, yet little is known about potential interactions between self-care and medications for chronic conditions in patients across Europe.

**Research questions:** 1. What are the potential interactions and frequencies of interactions between reported self-care items for common cold and medications for chronic conditions? 2. Which individuals are at higher risk for potential interactions?

**Methods:** This cross-sectional study was performed at 27 sites in 14 European countries. Participating sites distributed 120 questionnaires to 120 consecutive patients. 105 self-care practices were selected. Potential herb-drug and drug-drug interactions were analysed for the 10 most frequent items in the application mode intestinal absorption, teas and non-tea foodstuffs. Based on the potential number of interactions for each patient’s self-care, we analysed which sociodemographic characteristics are associated with a higher risk for interactions.

**Results:** The final analysis included 2724 patients, 62.5% were women; the mean age was 46.7 years (± 16.8). The total mean item utilization was 11.5 (± SD: 6.0, range: 0–53). Overall 39.9% had at least one of seven chronic conditions. The top 10 items in the application mode intestinal absorption have a potential risk for interaction in 87.3% of respondents, mean number of items used was 2.2 (± SD: 1.63). Among them, St John’s wort (used by 2.6% patients), ginger tea (13.5%), chamomile (17.5%), and peppermint (17.5%) have reported potential interactions with 14, five, four and three drugs, respectively. Three risk profiles were defined, and the group that uses three or more items with potential risk has significantly more females (66% versus 51.4%, *P* <0.001), is younger (44.23 ± 15.73 versus 49.89 ± 17.60 *P* <0.001) and more educated (13.46 ± 4.12 versus 11.61 ± 4.3 years, *P* <0.001).

**Conclusion:** This first cross-national study documented that self-care medications used for the common cold have a high potential of interaction with drugs for chronic conditions.

# Abstract: Importance of appropriate triage in patients with acute myocardial infarction with ST elevation in the ECG [Poster]

**DOI:** 10.1080/13814788.2017.1300653

**Published:** 2017-05-11

**Authors:** Marija Petek Šter

**Affiliations:** ^a^ Department for Family Medicine, University of Ljubljana Ljubljana Slovenia

## Abstract

**Background:** Acute myocardial infarction with ST elevation in the ECG (STEMI) is an emergency condition where rapid recognition of clinical priority and referral to primary coronary intervention (PCI) is crucial for a favourable clinical outcome. Triage in the emergency department is the first step of clinical workup. The Manchester triage system (MTS) is currently being used in Slovenia as the national triage system.

**Research question:** Does under-triage in patients with STEMI prolong time to PCI?

**Methods:** Retrospective analyses of data for 156 patients with STEMI admitted to the prehospital emergency department in the timeframe between 1 September 2012 and 31 August 2015 was undertaken. The patients were divided into two groups according to their MTS priority label: the group of appropriately triaged patients (red or orange priority) and the group of under-triaged patients (yellow and green priority). Comparison of system delay was taken into consideration and analysed in both groups.

**Results:** Among 156 analysed STEMI patients with the median age of 67.3 years (SD: 13.4 years), there were 96 (61.5%) men. There were 120 patients (76.9%) appropriately triaged (red or orange priority). Appropriately triaged patients received PCI 59.2 min quicker (86.2 min versus 145.4 minutes, *P* <0.001).

**Conclusion:** Proper triage category of STEMI patients is important because inappropriate triage category prolongs system delay (timeframe for PCI referral). ECG should be incorporated in triage algorithm: »chest pain«.

# Abstract: First results of the BeoNet registry: COPD [Poster]

**DOI:** 10.1080/13814788.2017.1300653

**Published:** 2017-05-11

**Authors:** Heidrun Lingner, Anna Nickel, K. Krüger, I. Aumann, M. Wacker, J.M. von der Schulenburg

**Affiliations:** ^a^ Hannover Medical School, Centre for Public Health and Healthcare Hannover Germany

## Abstract

**Background:** Healthcare research needs real-life-data, but they are scarce. The BeoNet registry (‘Beobachtungspraxen-Netz-register’) compiles the complete information from electronic patient records (eR) of primary care practices (GPs/pneumologists) and links them with patient-reported outcomes. First use case: assessment and analysis of information about patients with chronic obstructive pulmonary disease (COPD).

**Research question:** Are there any differences in demographics or absences from work between patients with (COPD+) and without COPD (COPD–) or between the target-populations from the different sites or the literature reports? What impediments occur when working with data from eR?

**Methods:** Systems for real time pseudonymous routine data provision from GPs and pneumologists from Hanover, Munich and Heidelberg were set up and tested. eR data, including diagnoses, treatments, procedures and medication were transferred via a standardized secure interface and compiled for analysis. The results of paper-based health-related quality of life questionnaires were linked to eR information. First descriptive analyses assessed the incidence, age, sex and comorbidities of COPD + and COPD– and their respective absences from work. Literature searches provided relevant publicly available data.

**Results:** Currently the weekly updated database (DB) contains 98 409 patient-IDs (female: 54%). The BeoNet-incidence of COPD is 4.5% (4455 IDs). Our results demonstrate that COPD patients in primary care are older (COPD+: 69.12%; COPD–: 52.4%) and more often males (49.56%) compared to patients without COPD (45.7%), are absent from work more frequently and most of them have more comorbidities as COPD–. Half of COPD + have three comorbidities (median), compared to two for COPD–; and on average five additional illnesses, compared to four for COPD–.

**Conclusion:** The demographics fit well with the literature, supporting the reliability of the registry data. Further research will analyse the additional information on the quality of life of COPD + and subgroup specific comorbidities correlations.
